# Current progress of ferroptosis study in ovarian cancer

**DOI:** 10.3389/fmolb.2022.966007

**Published:** 2022-08-26

**Authors:** Zhuomin Tan, Hui Huang, Wenyan Sun, Ya Li, Yinnong Jia

**Affiliations:** Department of Pharmaceutical Sciences, School of Pharmaceutical Sciences and Yunnan Key Laboratory of Pharmacology for Natural Products, Kunming Medical University, Kunming, China

**Keywords:** ovarian cancer, ferroptosis, ROS, System-Xc^−^, FSP1-CoQ10-NAD (P) H, GCH1–BH4, inducer, inhibitor

## Abstract

Tumors are the leading cause of death all over the world, among which ovarian cancer ranks the third in gynecological malignancies. The current treatment for ovarian cancer is liable to develop chemotherapy resistance and high recurrence rate, in which a new strategy is demanded. Ferroptosis, a newly discovered manner of regulatory cell death, is shown to be induced by massive iron-dependent accumulation of lipid reactive oxygen species. With the in-depth study of ferroptosis, its associated mechanism with various tumors is gradually elucidated, including ovarian tumor, which probably promotes the application of ferroptosis in treating ovarian cancer. To this end, this review will focus on the history and current research progress of ferroptosis, especially its regulation mechanism, and its potential application as a novel treatment strategy for ovarian cancer.

## 1 Introduction

Malignant tumors, one of the major diseases that seriously endanger human health, are the leading cause of death and a major public health problem all over the world. The treatments for tumors commonly include surgical therapy, radiotherapy, and drug therapy (chemotherapy), which is referred to as systemic therapy.

Ovarian cancer ranks the third in incidence and the second in mortality rate among gynecological malignant tumors worldwide. Meanwhile, it is one of the three most diagnosed malignant tumors in the female reproductive system. As diagnosed, 90–95% of ovarian cancers are primary ovarian malignant tumors and 5–10% are primary metastatic ovarian malignancy detected at other proximal sites. Among primary ovarian malignant tumors, epithelial cutaneous ovarian cancer is most commonly observed, accounting for more than 90% (Brown et al., 2014), in which high-grade serous cancer (high-grade serous carcinoma, HGSC) accounts for 70% (Re id et al., 2017). Most ovarian cancer patients do not display specific symptoms at early onset, and 75% are identified as advanced stage at the time of diagnosis (Lheureux et al., 2019). In addition, most of them exhibit a poor prognosis because of drug resistance and adverse effects. Therefore, it remains a great challenge to realize early diagnosis and overcome drug resistance in the current investigation of ovarian cancer.

At present, comprehensive treatment in ovarian cancer is frequently adopted, that is, the initial surgical treatment, supplemented by chemotherapy with the diagnosis and treatment has entered the era of accurate and individualized comprehensive disease management. At present, surgery is not only the most effective treatment to remove tumors but also a necessary method to confirm the diagnosis and specific stages by pathological analysis. Ovarian cancer patients at an early stage are treated with comprehensive staging surgery in order to completely remove the tumor and identify the specific stages, and patients at the advanced stage are treated with debulking surgery to remove tumor maximally (China Anti-Cancer Association Gynecological Cancer Professional Committee, 2021). Owing to the spread of most ovarian cancer in the early stage, surgery can barely remove all malignant lesions. In addition, the tumor volume of some advanced patients has decreased significantly after drug treatment, which facilitates surgery. Therefore, systemic chemotherapy has become the most important auxiliary therapy for ovarian cancer. At present, the first-line chemotherapy regimen for ovarian cancer is the administration of platinum drugs combined with paclitaxel, and the targeted drugs bevacizumab and PARP inhibitor are usually used for the maintenance of treatment. In recent years, the neoadjuvant chemotherapy (NACT) (van Driel et al., 2018) such as immunotherapy which utilizes inhibitors of immune examination point has become research hotspot, and gradually participates in ovarian cancer treatment.

In recent years, a new type of regulatory cell death (RCD) has been reported, somewhere between cell necrosis and apoptosis (Luo et al., 2021), which was first named ferroptosis in 2012. The cellular accumulation of lipid peroxides depending on the existence of iron eventually leads to cell ferroptosis. Cells that die in this way displayed unique characteristics, including increased mitochondrial membrane density, decreased or disappeared mitochondrial cristae, and reduced mitochondrial volume. Furthermore, ferroptosis has been reported to be associated with tumors, neurological diseases, kidney injury, and other diseases (Li et al., 2020). However, current studies of the regulatory mechanism of ferroptosis, such as the Fenton reaction, reactive oxygen species (ROS) regulation mechanism, System-Xc^−^-GSH-GPX4 pathway, *p53*-related pathway, FSP1-COQ10-NAD (P) H pathway, Hippo pathway, and GCH1–BH4 pathway, are not enough. At present, research reports of ferroptosis in ovarian cancer are less than 100, including the cell iron level, transsulfuration pathway, and Hippo pathway with participating genes such as *p53*, SCD1, and FZD7. Some publications also explored the application of ferroptosis in treating patients with platinum and paclitaxel resistance. In the future, more targets and drugs related to ferroptosis are expected for the diagnosis, treatment, and prognosis of ovarian cancer. Therefore, we summarized the role of ferroptosis in tumors, especially ovarian cancer, to provide a new research direction and novel treatment strategy.

### 1.1 Research history of ferroptosis

In 2001, during the study of the role of glutamate oxidative toxicity on nerve cell death, researchers found that dead nerve cells presented features of cell necrosis and apoptosis and were prevented by macromolecular synthesis inhibitors. However, this type of death depends on the generation of oxidative stress, different from the classical mode of cell death, and is therefore named oxidative death (Tan et al., 2001). In 2003, Dolma et al. (2003) reported that when they were screening for genetically selective antitumor small molecule drugs, they identified a new compound (named erastin). They found cells treated with erastin did not induce DNA fragmentation, with no change in nuclear morphology observed, and this treatment was irreversible on cells. Therefore, researchers believe that erastin-induced a new nonapoptotic cell death. Then, in 2008, Yang and Stockwell (2008) used the oncogene RAS as a specific mutation in artificial lethal screening, and between the two new compounds identified (named RSL5 and RSL3), RSL3 can activate erastin-mediated cell death mechanisms and can be inhibited by iron complexes. In 2012, Dixon et al. (2012) defined ferroptosis for the first time when they found that the small molecule erastin in the cancer-causing RAS-selective lethal compound triggered a form of nonapoptotic cell death. This is iron-dependent, which is different from apoptosis, programmed necrosis, and other known forms of cell death, and is usually accompanied by a massive accumulation of ROS, oxidative stress). It was also found that erastin inhibited cellular uptake of cystine by targeting the cystine/glutamate reverse transporter, thereby reducing the synthesis of reduced glutathione (GSH) and depleting GSH, which can be inhibited by the lipophilic antioxidant small molecule iron statin (ferrostatin-1, Fer-1).

Studies have found that the deficiency of glutathione peroxidase 4 (GPX4) leads to the accumulation of lipid peroxidation and abnormal cell death, which was induced by oxidative stress and prevented by knocking down the AIF gene (Seiler et al., 2008). According to previous studies, GPX4 is widely expressed in mammals, belongs to one of the seven glutathione peroxidases in mammals, is the only known major antioxidant enzyme that directly reduces peroxidase in cell membranes and lipoproteins, and interacts with tocopherol (vitamin E) to inhibit lipid peroxidation (Yant et al., 2003). In 2014, GPX4 was selected by Yang et al. (2014) and was confirmed as a key regulator of ferroptosis activated by erastin and RSL3. Erastin deactivates GPX4 by exhausting GSH, leading to the production of cytoplasmic and lipid ROS, whereas RSL3 directly binds and inactivates GPX4, leading to ferroptosis, and can be inhibited by an RSL3 inhibitor, such as iron complexation (DFOM), a MEK inhibitor (U0126), and antioxidants. In 2015, Jiang et al. (2015) first reported a link between *p53* and ferroptosis. One year later, Yang et al. (2016) reported that peroxidation of polyunsaturated fatty acids (PUFAs) occurred under the regulation of phosphorylase G2 (PHKG2) and lipoxygenase, respectively. In 2017, Doll et al. (2017) identified acyl-CoA synthetase long-chain family member 4 (ACSL4) as a key determinant of ferroptosis sensitivity, which can be used as a predictive marker of ferroptosis in different cellular environments and is an essential raw material for the production of PUFAs. In the following year, Ingold et al. (2018) found that selenium is present in GPX4 as the amino acid selenocysteine at position 21 and plays an indispensable role in GPX4 activity.

**FIGURE 1:**
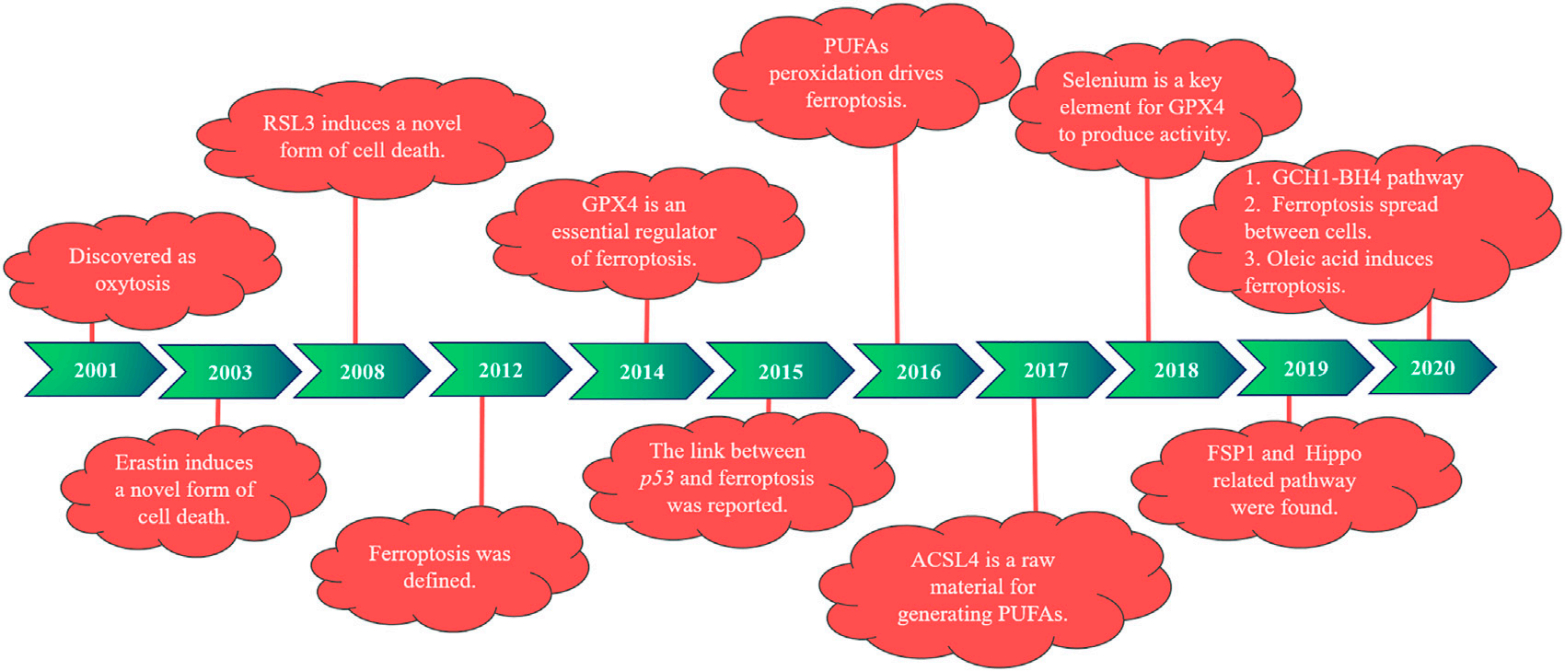
The development of ferroptosis at different time points.

Then, in 2019, Bersuker et al. (2019) and Doll et al. (2019) investigated the mechanism of ferroptosis suppressor protein 1 [FSP1, previously known as the apoptosis-inducing factor mitochondrial 2 (AIFM2)] in ferroptosis, identifying FSP1 as a potent ferroptosis resistance factor. At the same time, another group (Wu et al., 2019; Yang et al., 2019) found that cell density was a nongenetic factor that could modulate the sensitivity of ferroptosis by regulating the Hippo pathway effector YAP/TAZ, and more specific regulatory mechanisms were subsequently found in kidney and ovarian cancer studies. In 2020, a new pathway of GCH1–BH4 was discovered (Kraft et al., 2020; Wei et al., 2020). Riegman et al. (2020) reported that cell swelling was observed with ferroptosis, suggesting that it is an osmotic process that can be prevented by the administration of osmoprotectants. Ferroptosis was also found to distribute between cells in a lipid peroxidation and iron-dependent manner, which was not prevented by treatment with osmoprotectants. It has also been shown that oleic acid in lymph protects melanoma cells from ferroptosis in an acyl-CoA synthetase long-chain family member 3 (ACSL3)-dependent manner and can increase their ability to form metastatic tumors (Ubellacker et al., 2020). The history of ferroptosis development is also demonstrated in Figure 1.

## 2 Regulatory mechanisms of ferroptosis

**FIGURE 2:**
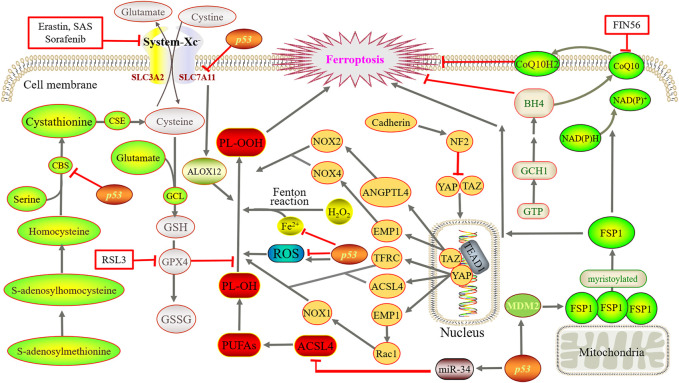
Main regulatory mechanisms of ferroptosis. Iron directs the Fenton reaction and induces large accumulation of ROS. Cystine is converted to cysteine for the synthesis of GSH. Then, GPX4 converts GSH to GSSG and reduces PL-OOH to PL-OH, thereby blocks the lipid peroxidation chain reaction. Using methionine as a sulfur donor, intermediate homocysteine, serine and cystathionine are converted into cysteine by the catalysis of CBS and CSE, simultaneously producing GSH. YAP and TAZ are abnormally activated, and bind to the TEAD after dephosphorylation. GCH1 overexpression promotes the generation of BH4. FSP1 oxidizes the terminal octadecylation modification of protein N to mediate lipid peroxidation. Simultaneously, FSP1 catalyzes the regeneration of ubiquinone and enters the circulation. By suppressing TFR1, ZIP14, ACSL4, SLC7A11 and CBS, *p53* reduce or increase ROS as well as transactivate MDM2.

In this section, the key regulatory pathways and mechanisms of proteins involved in ferroptosis are elucidated (Figure 2), with an emphasis on the valuable targets for treatment.

### 2.1 Iron reacts with Fenton

As the name suggested, the occurrence of ferroptosis depends on the existence of iron, which is essential for the normal maintenance of mammalian cells. Ingested iron absorbed by duodenal epithelial cells and red blood cell lysis produce Fe^2+^, and then, ceruloplasmin oxidizes Fe^2+^ to Fe^3+^. Fe^3+^ forms a binding substance with transferrin (TF), which is then transported through transferrin receptor 1 (TFR1) on the cell membrane surface and then endocytosed into cells. Later, Fe^3+^ is detached from the TF of the endosome and is finally reduced to Fe^2+^ by six-transmembrane epithelial antigen of prostate 3 (STEAP3) and then transported to the cytoplasm through the divalent metal ion transporter 1 (DMT1). In addition, Fe^2+^ constitutes labile iron pool (LIP) in cells as free iron ions, most of which are transferred to the mitochondria to participate in the formation of iron-dependent protein complexes, iron-sulfur clusters, heme, and other metabolic processes. However, heme oxygenase 1 (HO-1) catalyzes heme degradation to produce free iron, whose overexpression is able to accelerate erastin-induced ferroptosis. On the other hand, if Fe^2+^ is in excess in cells, it is stored as ferritin and released by the ferritin complex when needed or transported extracellularly by ferroportin 1 (FPN1) (Rockfield et al., 2017).

It is well known that iron deficiency often causes anemia, but excessive iron may imbalance iron homeostasis, thus driving the Fenton reaction to produce biotoxicity. Iron and hydrogen peroxide oxidize multiple substrates in biotoxic reactions called the Fenton reaction, which is able to produce hydroxyl radicals and higher oxidized states of iron (Winterbourn, 1995). The simplest representation of the Fenton reaction is as follows:

$${\text{Fe}}^{2+}+{\text{H}}_{2}{\text{O}}_{2}={\text{Fe}}^{3+}+\text{HO}\cdot +\text{HO}\cdot^{-}$$ (1)

Iron directs the Fenton reaction to that of the hydrogen peroxide (H_2_O_2_) generation of a hydroxyl radical (HO^**.**^). When GPX4 is inhibited, HO causes PUFAs to produce peroxidized lipids, and then, oxygen further catalyzes lipid oxidation and disrupts the membrane (Green, 2019). Peroxidized lipids can also produce new free radicals under the catalysis of iron, undergo a chain reaction, and promote the further propagation of lipid peroxidation (Conrad and Pratt, 2019).

### 2.2 Reactive oxygen species

ROS include superoxide anions, hydrogen peroxide, singlet oxygen, and hydroxyl radicals, which drive lipid peroxidation and kill cells by destroying lipids, proteins, and DNA (Lin et al., 2018). Tumor cell sustained growth and proliferation require “hypermetabolism,” and cells undergo metabolic reprogramming and abnormalities in mitochondrial function, eventually producing ROS and conducting proliferation signals to promote tumor development (Wang et al., 2019), but too much ROS may cause cell death. In this process, the mitochondrial electron transport chain produces electron leakage to generate a superoxide anion, NADPH oxidase on the cell membrane produces a superoxide anion, a superoxide anion forms H_2_O_2_ under the action of superoxide dismutase, and H_2_O_2_ is catalyzed by iron to produce HO, which further induces lipid peroxidation (large accumulation of ROS accumulation) and eventually leads to ferroptosis.

### 2.3 System-Xc^−^-GSH-GPX4 pathway

System-Xc^−^ is a heterodimeric amino acid reverse transporter that is widely distributed on the phospholipid bilayer. It is formed by solute carrier family members 11 (xCT) and solute carrier family 3 member 2 (SLC3A2, also called CD98hc) by disulfide bonds, also known as a cystine/glutamate reverse transporter. The former is a nutrient transporter protein that is frequently overexpressed in human malignancies (Koppula et al., 2018), and the latter uptakes extracellular cysteine while delivering equivalent amounts of glutamate to extracellular cells (Cao and Dixon, 2016). After cysteine enters the cell, cystine is converted to cysteine for the synthesis of GSH. Then, GPX4, which is active because of its selenocysteine content, converts GSH to oxidized GSH (GSSG) and reduces phospholipid hydrogen peroxide (PL-OOH) to phospholipid-alcohol (PL-OH), thereby blocking the lipid peroxidation chain reaction (Seibt et al., 2019). Studies have shown that erastin, sulfasalazine (SAS), and multiple kinase inhibitor sorafenib can block the function of xCT, deplete GSH, and thus induce ferroptosis (Gout et al., 2001; Dixon et al., 2012; Sun et al., 2016). However, the *p53* gene can downregulate cystine uptake by inhibiting SLC7A11 transcription, which limits intracellular GSH production (Yant et al., 2003).

In addition, studies have shown that cysteine, a nonessential amino acid, can be synthesized through the transsulfuration pathway in some mammalian cells and then binds glutamate to generate GSH under the action of glutamate cysteine ligase. Using methionine as a sulfur donor, intermediate homocysteine, serine, and cystathionine are converted into cysteine by the catalysis of cystathionine β-synthase (CBS) and cystathionine gamma-lyase (CSE), simultaneously producing GSH and the gas signaling molecule hydrogen sulfide (McBean, 2012; Sbodio et al., 2019). In addition, homocysteine can be derived from dietary methionine, which is converted to S-adenosine methionine and is then catalyzed by methyltransferase to produce S-adenosine and further generate homocysteine. In particular, CBS (also known as L-serine hydrolase) catalyzes the condensation of serine and homocysteine to form cystathionine. CSE (also known as L-cystathionine cysteine-lyase) is the only enzyme in mammals that can directly produce cysteine using the cystathionine generated by CBS.

### 2.4 *p53*-related pathway

The expression of *p53* in different species is highly conservative. Therefore, it is also called the guardian of genomes and cells, with a critical role. In 2015, it was reported by Jiang et al. (2015) for the first time that *p53* was related to ferroptosis, indicating its dual effect on ferroptosis as involving iron metabolism, lipid metabolism, amino acid metabolism, and ROS regulation. At present, ferroptosis can be classified into two main categories: GPX4-centered and *p53*-centered. As *p53* is a key regulator of cellular metabolism, it plays a vital role in ferroptosis regulation and is tightly related to ferroptosis initiation and progression (Liu and Gu, 2021; Liu and Gu, 2022). It is also related to the regulation mechanism in Section 2.1, Section 2.2, Section 2.3, and Section 2.5 of this review. It has been revealed that *p53* suppresses TFR1 and Zrt- and Irt-like protein 14 (ZIP14) to reduce cell iron intake and p53 can also be reduced or increased in different conditions (Liu and Gu, 2022). In addition, *p53* reduces ACSL4 by controlling miR-34 to suppress ferroptosis. On the one hand, *p53* can inhibit SLC7A11 and CBS to reduce the synthesis of GSH, ultimately inducing ferroptosis. On the other hand, it can promote the release of ALOX12 by reducing SLC7A11, which, in turn, causes lipid peroxidation and finally ferroptosis. Furthermore, *p53* can transactivate mouse double minute two homolog (MDM2), which activates FSP1 to promote ferroptosis.

### 2.5 FSP1-CoQ10-NAD (P) H pathway

GPX4 has long been recognized as a core regulatory protein that suppresses the occurrence of ferroptosis. In 2019, Bersuker et al. (2019) and Doll et al. (2019) reported FSP1 as an important ferroptosis regulator protein and confirmed that the FSP1-CoQ10-NAD (P) H pathway was independent of System-Xc^−^-GSH-GPX4 pathway and cooperated with it to inhibit ferroptosis lead by lipid peroxidation. Using expression cloning methods, AIFM2 was identified by Doll’s group. This unknown inhibitory gene of ferroptosis was therefore renamed FSP1, which was originally described by Wu et al. (2002) as a proapoptotic gene. FSP1 is primarily attached to the outer mitochondrial membrane, and when octadecylation occurs, FSP1 moves to the plasma membrane.

FSP1 inhibition of ferroptosis is mediated by ubiquinone (CoQ10), which utilizes the terminal octadecylation modification of protein N to target cytoplasmic membrane, as an NADPH-dependent CoQ10 induces the redox function to cause oxidation or reduction. The oxidizing CoQ10 is a lipophilic radical trapper and functions as an antioxidant to inhibit lipid peroxidation to avoid further ferroptosis. The reduction reaction generates panthenol, which can capture free radicals and can further mediate lipid peroxidation to inhibit ferroptosis. At the same time, FSP1 catalyzes the regeneration of ubiquinone by NAD (P) H and takes the circulation. Studies also found (Shimada et al., 2016) that by binding and activating squalene synthase, small molecule compound FIN56 inhibits the methoxyeric pathway synthesis of CoQ10, which causes the consumption of CoQ10 and induction of ferroptosis.

### 2.6 GCH1–BH4 pathway

It was reported that recently, a new pathway (the GCH1–BH4 protection pathway) was identified, which was parallel with and independent from the two classical pathways, namely, System-Xc^−^-GSH-GPX4 and FSP1-CoQ10-NAD (P) H (Li L. et al., 2021). By increasing its expression, CoQ10 can be elevated to slow down the progression of ferroptosis (Kraft et al., 2020). GTP cyclohydrolase 1 (GCH1), originating from GTP, is able to remove lipid peroxidation, and its overexpression promotes the generation of tetrahydrobiopterin (BH4) and avoids RSL3-induced ferroptosis (Wei et al., 2020).

## 3 Application of ferroptosis in ovarian cancer

### 3.1 Elevated iron levels

RCD plays a key role in the normal growth and development as well as maintaining homeostasis of multicellular organisms. Ferroptosis is a unique type of RCD, and many studies have demonstrated the relationship of ferroptosis was closely with tumor treatment. In particular, elevated iron levels are associated with the occurrence of various malignant tumors such as stem cell cancer, lung cancer, ovarian cancer, and renal cell cancer (Toyokuni, 2009; Xia et al., 2019). Studies have shown a soluble molybdenum compound called sodium molybdate that induced the elevation of LIP in ovarian cancer cells (Mao et al., 2022). In the tumor tissue of HGSC patients, the iron efflux pump ferroportin (FPN) decreases whereas TFR1 and TF increase. As a result, the intracellular level of iron is increased (Basuli et al., 2017). A similar situation was observed in genetic models of ovarian cancer tumor-initiating cells, suggesting that tumor cell intracellular iron levels are also elevated in the early stages of ovarian cancer. In a recent study, GPX4 knockout reduced iron levels and decreased interleukin-6 and tumor necrosis factor expression in ovarian cancer cells (Li D. et al., 2021). It suggests that we probably could interfere with the iron metabolism of tumor cells in the early stage of ovarian cancer to kill tumor cells.

### 3.2 Genes

#### 3.2.1 *p53* gene

The *p53* gene expresses the p53 protein, which acts as a DNA-binding transcription factor and selectively regulates the expression of certain *p53* transcriptional target genes. Studies have shown (McBean, 2012) that the inhibition of SLC7A11 expression reduced cystine uptake and sensitized ovarian cancer tumor cells to ferroptosis. However, *p53* ^3KR^ as an acetylation-deficient mutant of the *p53* protein fails to induce cell cycle arrest, senescence, and apoptosis, which completely retains the ability to regulate SLC7A11 expression and induce ferroptosis in tumor cells under oxidative stress. Furthermore, PARP inhibitors can inhibit SLC7A11 expression in a *p53*-dependent manner (Hong et al., 2021). Quartuccio et al. (2015) studied a mouse model of tubal epithelial cells with *p53* mutation, and the results showed that single *p53* mutation only migrated normal cells and normal cells transformed into tumor cells when combining *p53* mutation with the activation of K-ras. The expression of the *p53* gene also promotes ferroptosis induced by superparamagnetic iron oxide (SPIO) with the incubation in human serum, which provides a theoretical basis for the development of iron nanomaterials as new tumor therapeutic drugs (Zhang Y. et al., 2021). According to the results, SPIO could also induce ferroptosis in human ovarian cancer stem cells by attenuated autophagy (Huang et al., 2020). Almost 96% of HGSC cases are detected with mutations in the *p53* gene. Therefore, the interference with *p53*-mediated metabolic regulation has a significant impact on the treatment of ovarian cancer.

#### 3.2.2 Stearyl-coenzyme A desaturase gene

Stearyl-coenzyme A desaturase (SCD) is also known as D9-fatty acyl-CoA desaturase, through which monounsaturated fatty acids are produced from saturated fatty acids. In humans, SCD includes two isoforms, namely, *SCD5* and *SCD1* (prevalent form). In ovarian cancer tumor cells, *SCD1* is highly expressed, and studies have shown that the inhibition or deletion of *SCD1* gene could induce apoptosis and ferroptosis. The coadministration of erastin with *SCD1* inhibitor A939572 in mice with ovarian cancer tumor cells was much more effective than that of a single drug (Carbone and Melino, 2019; Tesfay et al., 2019). In addition, treatment with the *SCD1* inhibitors MF-438, CAY10566, and A939572 increased the sensitivity of ovarian cancer cells to the ferroptosis inducers RSL3 and erastin (Wang et al., 2022a). Furthermore, *SCD1* can induce ferroptosis in cancer cells mediated by the Menin-MLL inhibitor MI-463 (Kato et al., 2020).

#### 3.2.3 *Frizzleed-7*

Overexpression of frizzleed-7 (*FZD7*) can activate the oncogenic factor P63, upregulate GPX4, and avoid ferroptosis in cells. The expression of *FZD7* was directly associated with the expression of the GSH metabolism-related genes, namely, GSS, GSR, GPX2, and IDH (Wang et al., 2021a). Therefore, it is applicable to explore whether *FZD7* could be used as a novel biomarker to evaluate the sensitivity of platinum-resistant ovarian cancer cells to ferroptosis.

In addition, the solute carrier family (SLC) is associated with inducing ovarian cancer tumor cells. The anesthetic lidocaine downregulates the expression of SLC7A11 in a dose-dependent manner by promoting the intracellular expression of microRNA-382-5p (miR-382-5p), and the inhibition of miR-382-5P blocked ferroptosis in ovarian cancer cells (Sun et al., 2021). The SNAI2 gene can directly bind to the SLC7A11 promoter to regulate SLC7A11 expression (Jin et al., 2022). The tumor suppressor miR-424-5p inhibits ferroptosis in ovarian cancer by targeting ACSL4 (Ma et al., 2021).

### 3.3 Transsulfuration pathway

Studies have confirmed that the high level of GSH (Nunes and Serpa, 2018) and the high expression of related proteases (Kigawa et al., 1998) are closely related to the chemotherapy resistance of ovarian cancer. Verschoor and Singh (2013) treated a model derived from the tetracycline-induced overexpression of the proto-oncogene ETS-1 in ovarian cancer cells in 2008 with xCT inhibitors and inhibitors of the transsulfuration pathway. It was found that the overexpression of ETS-1 was accompanied by an increase in intracellular GSH and a significant reduction of GSH levels in models treated with transsulfuration pathway inhibitors. It indicates that the transsulfuration pathway plays an important role in GSH synthesis in ovarian cancer cells. Then, Chakraborty et al. (2015) found that the expression of CBS was increased in epithelial ovarian cancer cells (EOC) and silencing the genes of CBS could inhibit the migration and invasion of EOC tumor cells. Liu et al. (2020) found that the antioxidant transcription factor nuclear factor erythroid 2-related factor 2 (NRF2) upregulates CBS expression in erastin-resistant cells but causes ferroptosis after RNAi knockdown. It shows that the cell activation of the reverse transsulfuration pathway by NRF2/CBS enhances erastin-induced ferroptosis resistance. Thus, we can further think about the potential of the transsulfuration pathway to treat ovarian cancer with ferroptosis inducers. In this pathway, cysteine deprivation induced ferroptosis in tumor cells of ovarian clear cell carcinoma (Novera et al., 2020).

### 3.4 Hippo pathway

The Hippo pathway, consisting of a range of protein kinases and transcription factors regulated by cell-to-cell contact and cell density, is highly conserved in the evolution of both lower and higher animals. It is a potent inhibitory mechanism in tumor progression involved (Sun and Chi, 2020; Wang et al., 2021b) in apoptosis as well as tumorigenesis, metastasis, and chemoresistance. Yes-associated protein 1 (YAP) and transcriptional coactivator with PDZ-binding motif (TAZ) are two major transcription coactivators of transcription enhancement-related domains (TEAD) in the Hippo pathway. They affect cell phenotypes that include promoting cell proliferation, self-renewal, and inhibiting apoptosis by regulating the expression of target genes. YAP/TAZ is considered a receptor of cell density. When cell density is low and cell-to-cell contact is limited, the Hippo pathway is turned off. YAP and TAZ are abnormally activated and translocated from the cytoplasm to the nucleus after dephosphorylation, where they bind to the transcription factor TEAD, trigger cell ferroptosis, and inhibit apoptosis. However, the opposite result occurs.

In epithelial malignant tumor cells, cadherin overexpression mediates high cell density-enhanced cell contacts that then activate the Hippo pathway through NF2 (also called merlin) tumor suppressor proteins, thereby inhibiting nuclear transposition and YAP activity, which ultimately inhibits sensitivity to ferroptosis. Among them, YAP promotes ferroptosis by upregulating ACSL4 and transferrin receptor (TFRC), which can be inhibited by the small molecule CA3 (Song et al., 2018). In head and neck cancer, overexpression of epithelial membrane protein 1 (EMP1) regulates the expression of Rac1 and NADPH oxidase 1 (NOX 1) through the Hippo-YAP pathway, further promoting RSL3-induced ferroptosis (Wang et al., 2022b). Endogenous glutamate was reported to inhibit the iron-death sensitivity (Zhang X. et al., 2021) of lung adenocarcinoma by inhibiting ADCY10-dependent YAP. However, in malignant kidney tumor cells, TAZ is highly expressed and regulates EMP1 expression, which then induces NADPH oxidase 4 (NOX4) to regulate ferroptosis.

**FIGURE 3:**
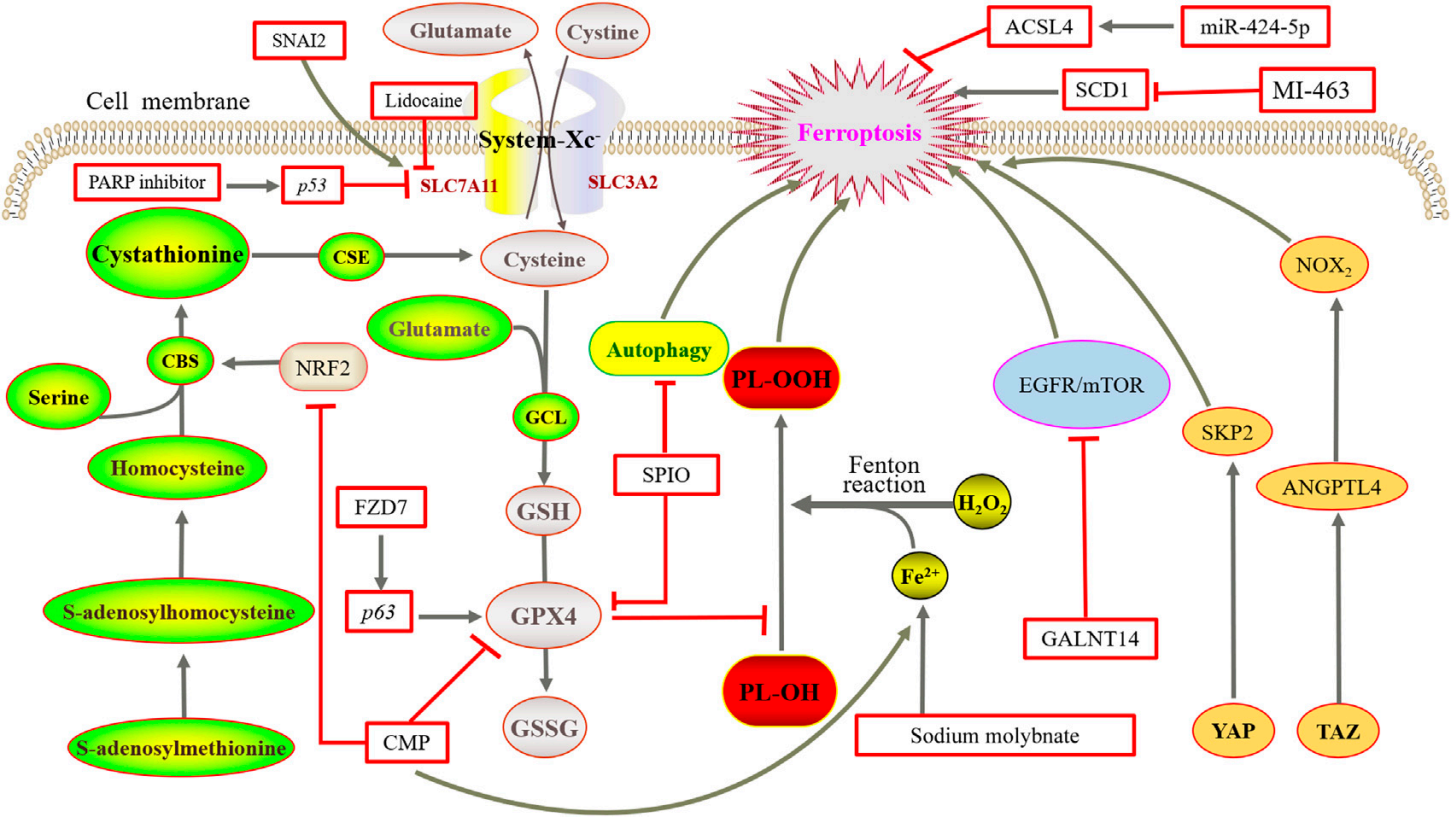
Mechanisms of ferroptosis in ovarian cancer. NRF2 upregulates CBS expression but causes ferroptosis after RNAi knockdown. Overexpression of FZD7 can activate the oncogenic factor *P63*, upregulate GPX4, and avoid undergoing ferroptosis. SPIO could also induce ferroptosis by attenuated autophagy. Sodium molybdate induced the elevation of LIP. The inhibition or deletion of SCD1 gene could induce ferroptosis. GALNT14 inhibits the EGFR/mTOR pathway, thereby downregulating ferroptosis. TAZ regulates the level of ANGPTL4 to manage the activity of NOX2, and finally leads to ferroptosis. YAP promotes ferroptosis by regulating SKP2.

It has also shown that the relatively high activity of YAP/TAZ promotes the invasion and metastasis of ovarian cancer cells and is often associated with poor prognosis (Yang and Chi, 2019). Cell density-dependent TAZ is the determinant factor of ferroptosis sensitivity in ovarian cancer. However, regulation is different from the pathway in kidney cancer. In ovarian cancer, TAZ regulates the level of its direct target gene angiopoietin-like 4 (ANGPTL4) to manage the activity of NADPH oxidase 2 (NOX2), finally leading to ferroptosis (Yang et al., 2020). YAP promotes ferroptosis in ovarian cancer malignant tumor cells by regulating its direct target gene, E3 ubiquitin ligase S phase protein-associated protein 2 (SKP2) (Yang W. H. et al., 2021). Furthermore, discoidin domain receptor tyrosine kinase 2 (DDR2) is upregulated in recurrent breast tumor cells, with sensitivity to ferroptosis enhanced by YAP/TAZ activation, which is unknown in ovarian cancer (Lin et al., 2021). In serous ovarian cancer, the TEAD family plays an important role, in which TEAD1 acts as a cotranscription factor in the Hippo pathway and the reduced TEAD2 expression may increase the accumulation of ROS, leading to ferroptosis (Ren et al., 2021). The currently studied proteins and genes of ferroptosis, especially in ovarian cancer, are summarized in Figure 3.

## 4 Ferroptosis-related drugs

At present, sorafenib is a United States FDA-approved ovarian cancer treatment agent that can induce ferroptosis (Sun et al., 2017). Other studies are still undergoing to develop new and effective ferroptosis-related drugs for ovarian cancer treatment.

### 4.1 Chemoresistance

The coadministration of platinum drugs with paclitaxel is the first-line drug treatment regimen for ovarian cancer. However, most ovarian cancer patients frequently develop chemoresistance, especially the resistance by taking platinum drugs, leading to recurrence. Platinum drugs are nonspecific drugs for affecting cell cycle that bind to DNA after entering tumor cells to form Pt–DNA complexes, producing intracellular ROS, which leads to cell damage and death. A multicenter phase 2 randomized controlled trial evaluated the oral coadministration of sorafenib with topotecan as a maintenance treatment and found a statistically and clinically significant improvement in progression-free survival in patients with platinum-resistant ovarian cancer (Chekerov et al., 2018). Micelles made from arachidonic acid-conjugated amphipathic copolymers targeting GPX4 after wrapping of RSL3 enhance the ability of RSL3 to induce ferroptosis, suggesting that chemotherapy resistance could be overcome by reducing the dosage of platinum drugs used and coadministration with this copolymer (Konstorum et al., 2020).

The ferroptosis inducer erastin acts with cisplatin synergistically by ROS-mediated and activated apoptosis to inhibit the growth of ovarian cancer cells, and it could be used as a sensitizer to overcome cisplatin resistance (Cang et al., 2020; Cheng et al., 2021). A combination of cisplatin with mTOR inhibitors causes an additive effect of cell death, and GALNT14, a member of the family of acetyl-galactosyltransferases, regulates the stability of EGFR proteins to inhibit the EGFR/mTOR pathway, thereby downregulating self-induced ferroptosis in ovarian cancer cells (Li H.W. et al., 2022). This suggests that GALNT14 provides a strategy to overcome cisplatin resistance in ovarian cancer patients. The MAP30 protein isolated from bitter melon also exhibits an effect on tumor treatment and can be used as a supplement to enhance the chemotherapy effect. It is a natural AMPK activator that induces ferroptosis consistent with cisplatin, enhancing the antitumor response and antichemoresistance in ovarian cancer (Chan et al., 2020).

Furthermore, several studies have demonstrated that the acquired synthesis of cysteine and GSH affects carboplatin resistance in ovarian cancer. Lopes-Coelho et al. (2016) found that hepatocyte nuclear factor 1β (HNF1) promotes glutathione synthesis to avoid carboplatin resistance in ovarian clear cell carcinoma. By studying ovarian cancer cell lines, including ES2, OVCAR3, OVCAR8, A2780, and A2780cisR, Nunes et al. (2018) clarified that cysteine protects cells from hypoxia and carboplatin effect to develop ovarian cancer. Santos et al. (2019) hypothesized that chemotherapy resistance of carboplatin could be reversed by disrupting cysteine metabolism. They used selenium-containing Chrysin (SeChry) as a competitive inhibitor of xCT, testing the effect of SeChry on three different ovarian cancer cell lines (ES2, OVCAR3, and OVCAR8) and two nonmalignant cell lines (HaCaT and HK2). The results showed that SeChry depleted GSH and inhibited CBS, preventing the production of the antioxidant hydrogen sulfide. Then, a new nanomedicine is designed to improve the specificity of drugs to tumor cells, which has achieved good effects in the treatment of ovarian cancer.

It was also shown that the upregulation of the drug efflux transporter ABCB1 easily led to relapse of resistance to docetaxel therapy, but the ovarian cancer cell cycle remained in the G2/M phase after coadministration with erastin, and the relapse of resistance was reversed (Zhou et al., 2019). Promyelocytic leukemia protein (PML) can increase lipid peroxidation, regulate mitochondrial metabolism and iron metabolism in tumor cells, and then enhance the chemosensitivity of HGSC (Gentric et al., 2019).

### 4.2 Other

Artemisinin (ARS) is a classic antimalarial drug and can fight tumors (Efferth, 2017). The cellular response of ARS and its derivatives to tumor cells involves multiple cell death modes, including apoptosis, autophagy, ferroptosis, and necrosis. Artesunate (ART), derived from *Artemisia annua* leaves, has potent antiproliferative and cytotoxic effects on ovarian cancer cells (Greenshields et al., 2017). The strong anticancer effects of ART include that low concentrations of ART lead to ROS-independent cell cycle arrest in the G1 phase and inhibition of mTOR signaling; high concentrations of ART lead to ROS production, mediated by apoptosis and ferroptosis.

Furthermore, the plant extract uronic acid can promote ferroptosis through the activation of the JNK/*p53* signaling pathway (Ruan et al., 2021). Carboxymethylated pachyman (CMP) inhibited HO-1 signaling, xCT, and GPX4, as well as upregulated Fe^2+^ expression by downregulating NRF2 expression, ultimately leading to the induction of ferroptosis in ovarian cancer cells. According to recent reports, sodium molybdate can modulate the production of nitric oxide to exhaust GSH, raise iron levels, and promote ferroptosis in ovarian cancer cells (Zheng et al., 2022). In the view of immunotherapy, it has found that checkpoint inhibitors induce CD8^+^ T cells to trigger ferroptosis in mice bearing ovarian tumor (Immunotherapy Activates, 2019). Furthermore, the combination of the Menin-MLL inhibitor MI-463 with the thioredoxin reductase inhibitor auranofin could synergistically increase the mortality of ovarian cancer tumor cells (Jing et al., 2022).

## 5 Discussion and perspective

Ferroptosis, discovered as a novel type of RCD, occurs in an iron-dependent manner after massive accumulation of ROS, presenting features different from other well known forms of cell death such as apoptosis and autophagy. The reported regulatory mechanisms of ferroptosis include the Fenton response, System-Xc^−^-GSH-GPX4 pathway, FSP1-CoQ10-NAD (P) H pathway, Hippo pathway, and GCH1–BH4 pathway. At present, most of these pathways have been studied extensively in malignant tumors, neurological diseases, and liver and kidney injury, except the latter two pathways, which are relatively rarely studied and showing no clear target or index for the treatment or prognosis in each disease. As a rising star, ferroptosis provides a new idea for the research of various diseases and plays a significantly important role in the remarkable success of tumor treatment. In recent years, a large number of studies have screened reliable mRNAs and genes to predict the prognosis of ovarian cancer patients by means of bioinformatics (Feng et al., 2022), comprehensive clinical analysis (Peng et al., 2021), and construction of ferroptosis-related gene models (Yang L. et al., 2021; Chen et al., 2021; Li X. X. et al., 2021; Zhang J. et al., 2021; Ye et al., 2021; You et al., 2021; Yu et al., 2021; Li Y. et al., 2022; Wang H. et al., 2022; Zou et al., 2022). It provides preciously applicable suggestions for exploring the therapeutic targets and prognostic indicators related to ferroptosis in ovarian cancer. At the same time, research of new compounds as inducers and inhibitors also provides a meaningful reference for the optimization of ovarian cancer treatment regimen. However, on the basis of the current research results, it is too soon to conclude the role of ferroptosis in the diagnosis, treatment, and prognosis of ovarian cancer for clinical purpose. In the future, inducers and inhibitors related to ferroptosis of ovarian cancer tumor cells, as well as the combined effects of new and classic drugs, the drug resistance of tumor cells, and the specific regulatory mechanisms, remain to be thoroughly studied.
